# A Time-Resolved PTR-ToF-MS Workflow for Characterizing Opening-Burst Volatile Features in Canned Antarctic Krill Under Equivalent F_0_ Processing

**DOI:** 10.3390/foods15172968

**Published:** 2026-08-24

**Authors:** Peizi Sun, Songyi Lin, Yuting Tan, Yajie Qin, Chen Tao, Dongmei Li

**Affiliations:** 1 School of Food Science and Technology, National Engineering Research Center of Seafood, Dalian Polytechnic University, Dalian 116034, China; 2 Engineering Research Center of Seafood of Ministry of Education of China, Dalian 116034, China; 3 Collaborative Innovation Center of Seafood Deep Processing, Dalian Polytechnic University, Dalian 116034, China; 4 SKL of Marine Food Processing & Safety Control, Dalian Polytechnic University, Dalian 116034, China

**Keywords:** volatile organic compounds (VOCs), post-opening headspace, odor plume, volatile release kinetics, retort sterilization, process comparison

## Abstract

Volatile release immediately after opening canned foods is highly transient and may not be adequately represented by equilibrium or quasi-equilibrium headspace measurements. In this study, time-resolved proton-transfer-reaction time-of-flight mass spectrometry (PTR-ToF-MS) was used to characterize opening-burst volatile features in canned Antarctic krill processed to an equivalent sterilization intensity (F_0_ ≈ 9 min). Raw krill, pretreated but non-retorted samples, and samples retorted using different temperature–time combinations were monitored online for 180 s after opening, with particular attention to the 0–60 s burst phase. Twenty-three of the twenty-five retained features reached their maximum responses within 10 s, including two within 5 s, showing that the dominant volatile changes occurred immediately after opening. The maximum background-corrected response during 0–60 s (C_max,0–60_) and the corresponding area under the response curve (AUC_0–60_) were used to describe transient release. The nine highest-ranked features accounted for 76.78% of the cumulative AUC_0–60_ response within the retained candidate feature set. Although the retort treatments had similar F_0_ values, their burst-phase fingerprints remained distinguishable. Sulfur-associated features varied markedly among treatments, whereas several low-molecular oxygenated features showed strong responses after the 121 °C high-temperature/short-time treatment. These findings show that the first seconds after opening contain substantial volatile-release information and that equivalent sterilization lethality does not necessarily produce the same opening-burst fingerprint. Time-resolved PTR-ToF-MS therefore enables direct examination of the initial post-opening volatile environment and comparison of different thermal processing routes. Chemical names are reported as putative annotations rather than definitive identifications.

## 1. Introduction

Antarctic krill (*Euphausia superba*) is a marine protein resource with high biomass and nutrient density and has considerable potential for value-added utilization [1]. Krill meat and krill oil have also attracted attention because of their nutritional value and potential health-related effects, supporting the development of krill-derived foods and other products [2]. Canning is one route for producing microbiologically safe and shelf-stable krill products. During processing, soaking, blanching, and retorting can alter volatile composition through precursor loss, heat-induced reactions, and changes in the food matrix, which in turn affect the release of volatile compounds [3]. Once a can is opened, volatiles accumulated in the headspace and released from the product surface disperse rapidly into the surrounding air, forming a short-lived odor plume. This initial release may contribute to the first aroma impression of the product [4]. Its rapid nature makes the temporal behavior of volatile release an important part of post-opening flavor characterization.

Studies of Antarctic krill flavor have mainly used headspace solid-phase microextraction coupled with gas chromatography–mass spectrometry (HS-SPME/GC–MS) and gas chromatography–ion mobility spectrometry (GC–IMS) to describe volatile composition and processing-related changes. Fan et al. [5] characterized volatile compounds in Antarctic krill by HS-SPME/GC–MS and reported a profile dominated by aldehydes, alcohols, and ketones. In our previous work, GC–IMS combined with partial least squares–discriminant analysis (PLS-DA) was used to characterize changes in the volatile fingerprint during canned Antarctic krill processing [6]. We subsequently examined the effects of sterilization intensity using GC–IMS and GC–MS at F_0_ values of 6, 9, 12, and 15 min and found that F_0_ ≈ 9 min provided a relatively favorable flavor profile compared with more severe sterilization conditions [7]. These chromatographic studies provide detailed information on volatile composition, but their analytical time scale differs from that of the opening event considered here. Headspace enrichment and chromatographic analysis generally require substantially longer than the few seconds in which the initial volatile burst occurs, so the original peak-and-decay sequence is not preserved [8]. SPME extraction can also represent volatile compounds differently depending on their physicochemical properties [9]. Characterizing the opening event therefore requires direct monitoring at a time resolution sufficient to follow the release sequence as it occurs.

Proton-transfer-reaction mass spectrometry (PTR-MS) uses soft chemical ionization, commonly with H_3_O^+^ reagent ions, for direct online detection of volatile organic compounds [10]. Its sub-second to second time resolution is well suited to rapidly changing aroma-release processes [11]. PTR-ToF-MS has been used in several food systems where temporal changes are important. Muñoz-González et al. [12] monitored post-oral and nasal volatile evolution to examine wine aroma persistence and inter-individual variation. Gonzalez-Estanol et al. [13] combined dynamic nosespace PTR-ToF-MS with temporal sensory methods to study aroma release and perception in chocolate–hazelnut spreads. Wieland et al. [14] used 1 Hz PTR-ToF-MS to monitor volatile changes in coffee-roasting exhaust. Seconds-scale volatile peaks and their subsequent decay have also been recorded after hot-water reconstitution of food products [15]. These applications show that PTR-ToF-MS is suitable for following rapid changes in volatile release.

However, the seconds-scale volatile event immediately after opening canned foods has received limited attention, particularly when thermal treatments are compared at equivalent sterilization lethality. Two questions were therefore addressed in the present study: (i) how volatile features change during the short period immediately after opening, and (ii) whether different temperature–time combinations producing similar F_0_ values also produce similar opening-burst fingerprints. Raw Antarctic krill, pretreated but non-retorted samples, and canned samples processed at 110, 115, 118, and 121 °C to F_0_ ≈ 9 min were analyzed by time-resolved PTR-ToF-MS. Signals were monitored continuously for 180 s after opening, with the 0–60 s period defined as the main burst phase. The maximum background-corrected response during 0–60 s (C_max,0–60_), the corresponding area under the response curve (AUC_0–60_), and the complete time profiles were used to describe transient release behavior and compare processing treatments. PTR-ToF-MS signals were interpreted mainly at the *m*/*z* feature and chemical-family levels; chemical names were used only as putative annotations where the available evidence supported such interpretation.

## 2. Materials and Methods

### 2.1. Materials

Frozen Antarctic krill (*Euphausia superba*) was provided by Dalian Liaoyu Fishery Group Co., Ltd. (Dalian, China). The raw material was harvested in May 2025 from Antarctic FAO Area 48.1, peeled on board, immediately frozen at −20 °C, and transported to the laboratory under cold-chain conditions in August 2025. Samples were portioned at 600 g per bag, packed in transparent food-grade PA/PE (nylon/polyethylene) composite vacuum bags (120 μm thickness), vacuum-sealed, and stored at −20 °C until use.

### 2.2. Preparation of Canned Antarctic Krill Samples

Canned Antarctic krill samples were prepared according to Sun et al. [7], with the pretreatment and F_0_-equivalent retort conditions used in the present study described below. Frozen krill meat from the same batch was thawed at 4 °C for 6 h. Visible impurities were removed, and the meat was rinsed with ultrapure water. The krill meat was then soaked in 2% (*w*/*w*) NaCl solution at 4 °C for 15 min at a solution-to-sample ratio of 2 mL/g.

After draining, fresh 2% (*w*/*w*) NaCl solution was preheated to 80 ± 2 °C. The krill meat was immersed in the preheated solution at a solution-to-sample ratio of 1 mL/g and blanched for 20 s, with timing started immediately upon immersion. The samples were then drained for approximately 2 min.

For canning, 74 g of pretreated krill meat and 36 g of 2% (*w*/*w*) NaCl brine were placed in each metal can, giving a net content of 110 g. The cans were exhausted at 100 °C for 10 min and vacuum-sealed at −0.04 MPa. Group B underwent the same pretreatment, filling, exhausting, and sealing procedures but was not retorted.

F_0_ was used as the common lethality metric for comparing the four retort schedules. For low-acid canned foods, *Clostridium botulinum* is commonly used as a reference organism for thermal-process design; its reported decimal reduction time at 121.1 °C is approximately 0.21 min. With z = 10 °C, the corresponding D value at 115 °C is approximately 0.855 min [16]. The target sterilization value was therefore set at F_0_ ≈ 9 min, referenced to T_ref_ = 121.1 °C and z = 10 °C. The accumulated sterilization value was calculated as:

(1) $$F_{0}=\int_{0}^{t}10^{(T-T_{ref})/z}\;dt$$

where F_0_ is the cumulative sterilization value (min), T is the measured can-center temperature at process time (°C), T_ref_ is the reference temperature (121.1 °C), z is the temperature increase required for a tenfold change in the decimal reduction time (10 °C), and t is the total process time (min).

**Figure 1:**
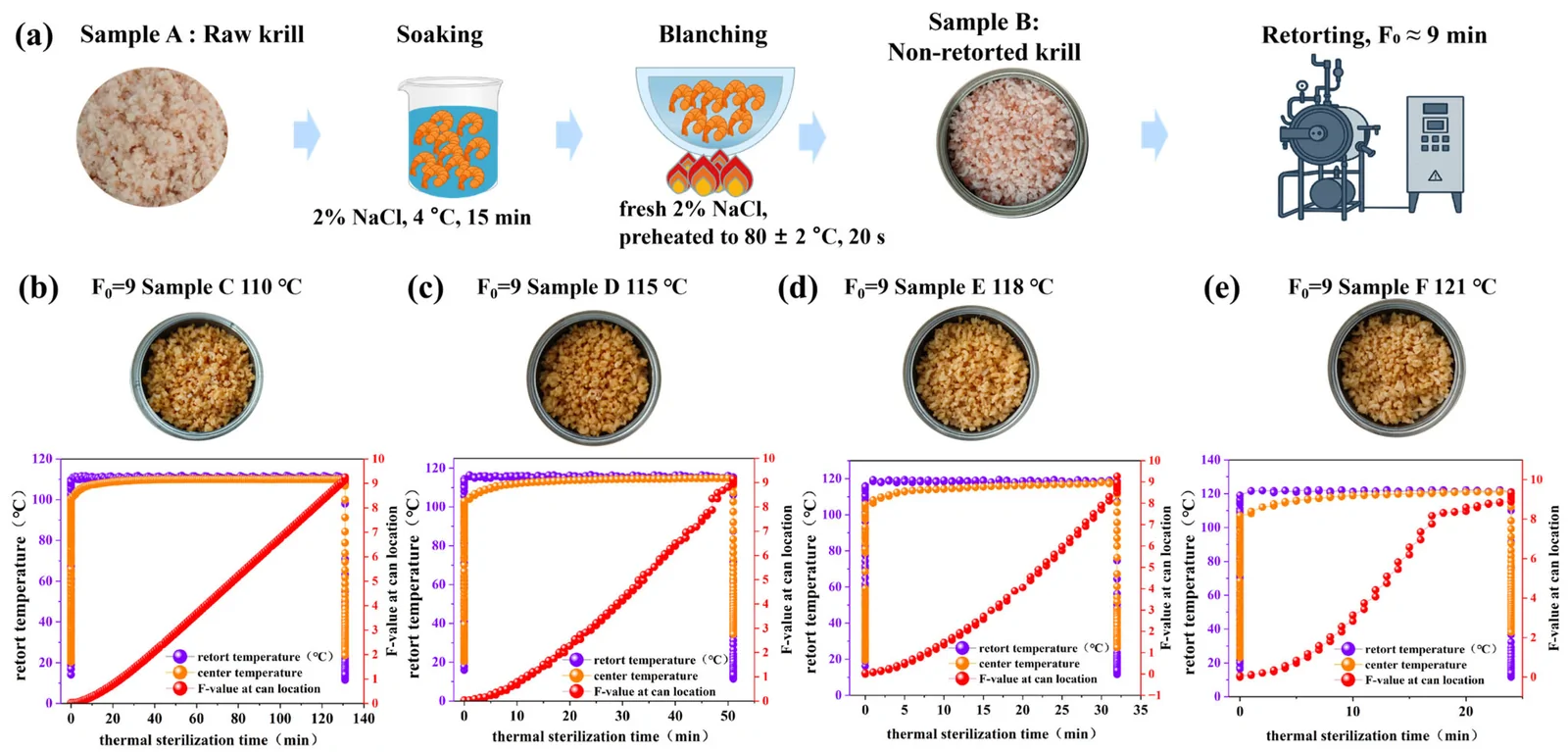
Sample grouping and F_0_-equivalent retort design for canned Antarctic krill. (**a**) Processing workflow and group definitions. Group A, raw krill meat without pretreatment; Group B, pretreated, filled, exhausted, and vacuum-sealed but non-retorted samples; Groups C–F, samples retorted at 110, 115, 118, and 121 °C, respectively, targeting F_0_ ≈ 9 min. The pretreatment procedure included thawing, rinsing, soaking in 2% (*w*/*w*) NaCl for 15 min, and blanching for 20 s in fresh 2% (*w*/*w*) NaCl solution preheated to 80 ± 2 °C, with timing initiated immediately upon immersion. (**b**–**e**) Can-center temperature profiles and cumulative F_0_ during the four retort schedules, 110 °C, 115 °C, 118 °C, and 121 °C, respectively. F_0_ was calculated from the complete can-center thermal history.

Groups C–F were processed in a counter-pressure steam retort (ZM-100; Guangzhou Biaoqi Packaging Equipment Co., Ltd., Guangzhou, China) using temperature–time combinations designed to achieve F_0_ ≈ 9 min: 110 °C for 116 min, 115 °C for 36.7 min, 118 °C for 18.4 min, and 121 °C for 9.2 min, respectively. Can-center temperature and cumulative F_0_ were recorded throughout the thermal process and are shown in Figure 1. After thermal processing, the cans were rapidly cooled to below 38 °C and held at 25 ± 1 °C for at least 7 days before PTR-ToF-MS analysis.

### 2.3. PTR-ToF-MS Analysis and Mass Spectral Data Processing

PTR-ToF-MS measurements were adapted from Ni et al. [17] for direct time-resolved sampling during can opening. Volatile features were monitored online without headspace enrichment. A heated PEEK tube (1/16″; inner diameter approximately 0.75 mm) fitted with a 0.2 μm PTFE droplet filter, both supplied by TOFWERK AG (Thun, Switzerland), was used as the sampling inlet. The inlet was positioned 1.5–2.0 cm above the center of the can opening. Airflow disturbance around the sampling area was minimized during measurement, and the sampling flow rate was maintained at 100 sccm.

Measurements were carried out using a PTR-ToF-MS 4000 instrument (IONICON Analytik GmbH, Innsbruck, Austria) operated in H_3_O^+^ mode. Time-of-flight data were acquired with TofDaq software (Version 1.99 r1369, TOFWERK AG, Thun, Switzerland), and peak extraction was performed using PTR-MS Viewer 3.4.3 (IONICON Analytik GmbH, Innsbruck, Austria). The transfer line and drift tube were maintained at 80 °C. Drift-tube pressure and voltage were 3.30 mbar and 600 V, respectively, corresponding to E/N = 125 Td. Spectra were recorded over *m*/*z* 20–350 at approximately 0.50 s per spectrum.

For each can, data acquisition began before opening and continued for 180 s after opening. The opening moment was defined as t = 0 s. The interval from −5 to 0 s was used as the can-specific pre-opening baseline, 0–60 s as the burst phase, and 60–180 s as the post-burst phase. Each treatment contained three independent cans (*n* = 3), and each can was measured only once because the opening event could not be repeated. Measurement order was randomized. Three instrumental blank measurements were also collected during each analytical run to monitor background contamination and instrumental stability. These measurements were separate from the −5 to 0 s baseline used for correction of each individual can.

Mass calibration in H_3_O^+^ mode used *m*/*z* 21.022 (H_3_^18^O^+^), *m*/*z* 203.943 (C_6_H_5_I^+^, a diiodobenzene-derived calibration ion), and *m*/*z* 330.848 (C_6_H_5_I_2_^+^, protonated 1,3-diiodobenzene). Time-of-flight spectra were processed using dead-time correction (20 ns; Poisson correction), reference-peak correction, and internal mass calibration. Mass accuracy after calibration was maintained within 5 ppm. Signals were converted to normalized counts per second (ncps) and normalized using the H_3_O^+^ reagent-ion signal and drift-tube operating parameters.

Peak extraction was performed using the Auto Search function with smoothing = 5 and threshold = 10. Isotopic peaks, reagent-ion/water-cluster-related signals, and interfering signals at *m*/*z* 21, 30, 32, 37, and 55 were excluded. Features with S/N ≥ 3 were considered detectable. A feature was retained when it was detected in at least two of the three independent cans within a treatment and had a within-treatment RSD < 30% [18]. Can-specific baseline correction and subsequent time-domain calculations are described in Section 2.4.

For semi-quantitative analysis, ncps values were converted to approximate mixing ratios in ppbv using the Lindinger relationship. Compound-specific reaction rate constants (K_R_) were used where available; otherwise, K_R_ = 2.0 × 10^−9^ cm^3^ s^−1^ was applied [19]. Because PTR response factors differ among ions, the calculated ppbv values were treated as semi-quantitative feature responses and were used mainly to compare the same *m*/*z* feature among treatments. Differences among separate *m*/*z* features and AUC-based rankings therefore represent relative PTR-ToF-MS responses rather than absolute differences in chemical abundance.

PTR-ToF-MS does not provide chromatographic separation, and retention indices are therefore not applicable. Feature interpretation was based on measured *m*/*z*, plausible ion formulae, PTR ion chemistry, and literature information. Where the evidence supported a plausible assignment, names were reported using the “-like” designation. Features that could not be assigned with sufficient structural specificity were reported at the formula or chemical-family level, or as unassigned *m*/*z* features. Possible isomers, fragments, adducts, isobaric species, and other unresolved contributions were not assigned to a unique structure on the basis of PTR-ToF-MS data alone [20].

### 2.4. Time-Series Processing and Burst-Phase Descriptors

Time-resolved volatile features obtained during can opening were processed using the semi-quantitative ppbv time series described in Section 2.3. Each treatment consisted of three independent cans (*n* = 3), and each can was measured only once. PTR-ToF-MS spectra were acquired continuously at a time resolution of approximately 0.50 s. For visualization, representative data points at 5 s intervals were displayed in selected figures; however, all time-domain descriptors were calculated from the complete continuous time series without temporal down-sampling. The data were organized as “feature (*m*/*z*)–time–sample ID (independent can)”. For the equations below, $C_{r}(t)$ denotes the semi-quantitative response (ppbv) of a given *m*/*z* feature at time t (s) in the r-th independent can (r=1,2,3).

(1) Pre-opening baseline and baseline correction

For each independent can, the mean response during the −5 to 0 s pre-opening window was used as the can-specific baseline:

(2) $$C_{base,r}=\frac{1}{5}\int_{-5}^{0}C_{r}(t)\;dt$$

where $C_{base,r}$ is the mean pre-opening baseline response (ppbv).

The baseline-corrected response was then calculated as:

(3) $$C_{corr,r}(t)=\max[C_{r}(t)-C_{base,r},0]$$

where $C_{corr,r}(t)$ is the baseline-corrected response (ppbv). Negative values after baseline subtraction were set to zero.

(2) Maximum burst-phase response

The maximum baseline-corrected response during the 0–60 s burst phase was defined as C_max,0–60_:

(4) $$C_{max,0-60,r}={max\;}_{t\in [0,60]}\;C_{corr,r}(t)$$

where C_max,0–60,r_ is the maximum opening-induced response (ppbv) observed in the r-th can during the burst phase.

For descriptive presentation in Table 1, the corresponding uncorrected maximum response over 0–60 s was also recorded as the raw C_max,0–60_. The baseline-corrected value in Equation (4), rather than the raw maximum, was used for time-domain interpretation.

(3) Cumulative burst-phase response

The cumulative response during the 0–60 s burst phase was expressed as ${\mathrm{AUC}}_{0-60}$. Numerical integration was performed using the trapezoidal rule on the complete time series:

(5) $${\mathrm{AUC}}_{0-60,r}=\underset{i=1}{\overset{N-1}{\sum }}\frac{C_{corr,r}(t_{i})+C_{corr,r}(t_{i+1})}{2}(t_{i+1}-t_{i})$$

where $t_{i}$ and $t_{i+1}$ are consecutive acquisition times (s), N is the number of data points between 0 and 60 s, and ${\mathrm{AUC}}_{0-60,r}$ is expressed as ppbv·s.

(4) Time to maximum response and baseline-stability screening

The time at which the maximum baseline-corrected response occurred within the burst phase was recorded as $t_{max,0-60}$:

(6) $$t_{max,0-60,r}=\underset{0\leq t\leq 60}{\arg\;\max}C_{corr,r}(t)$$

where $t_{max,0-60,r}$ is expressed in seconds.

For time-domain screening, only features showing a positive opening-induced response and a pre-opening baseline RSD ≤ 30% were retained in the candidate feature set Ω. Baseline stability was evaluated from the $C_{base,r}$ values of the three independent cans within each treatment. These criteria were applied after the detection and reproducibility screening described in Section 2.3.

(5) AUC-based feature ranking and cumulative contribution

For each retained feature, AUC_0–60_ was first averaged across the three independent cans within each treatment. The treatment-level means were then averaged across the six groups to obtain the overall mean AUC_0–60_ used for feature ranking.

The relative contribution of feature j to the cumulative response of the retained candidate feature set was calculated as:

(7) $${\mathrm{Contribution}}_{j}(\%)=\frac{{\bar{\mathrm{AUC}}}_{0-60,j}}{\underset{k\in \Omega }{\sum }{\bar{\mathrm{AUC}}}_{0-60,k}}\times 100\%$$

where ${\bar{\mathrm{AUC}}}_{0-60,j}$ is the overall mean ${\mathrm{AUC}}_{0-60}$ of feature j, and Ω denotes the retained candidate feature set. Features were ranked in descending order of ${\bar{\mathrm{AUC}}}_{0-60}$. The cumulative contribution of the nine highest-ranked features was obtained by summing their individual contributions. The AUC-based contribution represents the relative share of the measured semi-quantitative PTR-ToF-MS feature response within the retained candidate set. It does not represent absolute chemical abundance or sensory contribution.

### 2.5. Statistical Analysis

Each treatment consisted of three independent cans (*n* = 3), with the individual can treated as the experimental unit. Unless otherwise stated, descriptive values are reported as arithmetic means of the three independent cans. For burst-phase fingerprint analysis, the mean background-corrected response over 0–60 s (C_corr,0−60_) was calculated for each feature in each independent can. The resulting feature × sample matrix was Z-score-standardized before hierarchical clustering and heatmap visualization. Partial least squares–discriminant analysis (PLS-DA) was used for exploratory assessment of treatment-related patterns. Model performance was evaluated using cumulative R^2^X, R^2^Y, and Q^2^ values, together with a 200-iteration permutation test. Features with variable importance in projection (VIP) values > 1 were retained for feature screening. Pearson correlation coefficients were calculated across all 18 independent cans using C_corr,0−60_ values, followed by hierarchical clustering of the correlation matrix. Correlations were interpreted as patterns of co-variation rather than evidence of causal relationships. Pearson correlation analysis was performed using SPSS Statistics 18.0 (SPSS Inc., Chicago, IL, USA). PLS-DA and related multivariate analyses were performed using SIMCA-P v13.0 (Umetrics, Malmö, Sweden) and MetaboAnalyst 5.0 (McGill University, Montreal, QC, Canada). Figures were prepared using OriginPro 2024 (OriginLab Corporation, Northampton, MA, USA).

## 3. Results and Discussion

### 3.1. F_0_-Equivalent Processing Design and Opening-Induced Volatile Release Kinetics

The six sample groups represented successive processing stages and different retort temperature–time combinations (Figure 1a). Group A was raw Antarctic krill meat without pretreatment. Group B underwent soaking, blanching, filling, exhausting, and vacuum sealing but was not retorted. Groups C–F were retorted at 110, 115, 118, and 121 °C, respectively, using the time schedules described in Section 2.2. Although the retort temperatures differed substantially, the corresponding thermal processes produced similar accumulated lethality, with F_0_ values close to 9 min. The can-center temperature and cumulative F_0_ profiles are shown in Figure 1b–e. These profiles confirm that the four thermal treatments provided comparable sterilization intensity while following clearly different temperature–time histories. Visible changes also occurred across the retorted samples. Compared with the non-retorted groups, the thermally processed krill showed progressive darkening as retort temperature increased [20].

**Figure 2:**
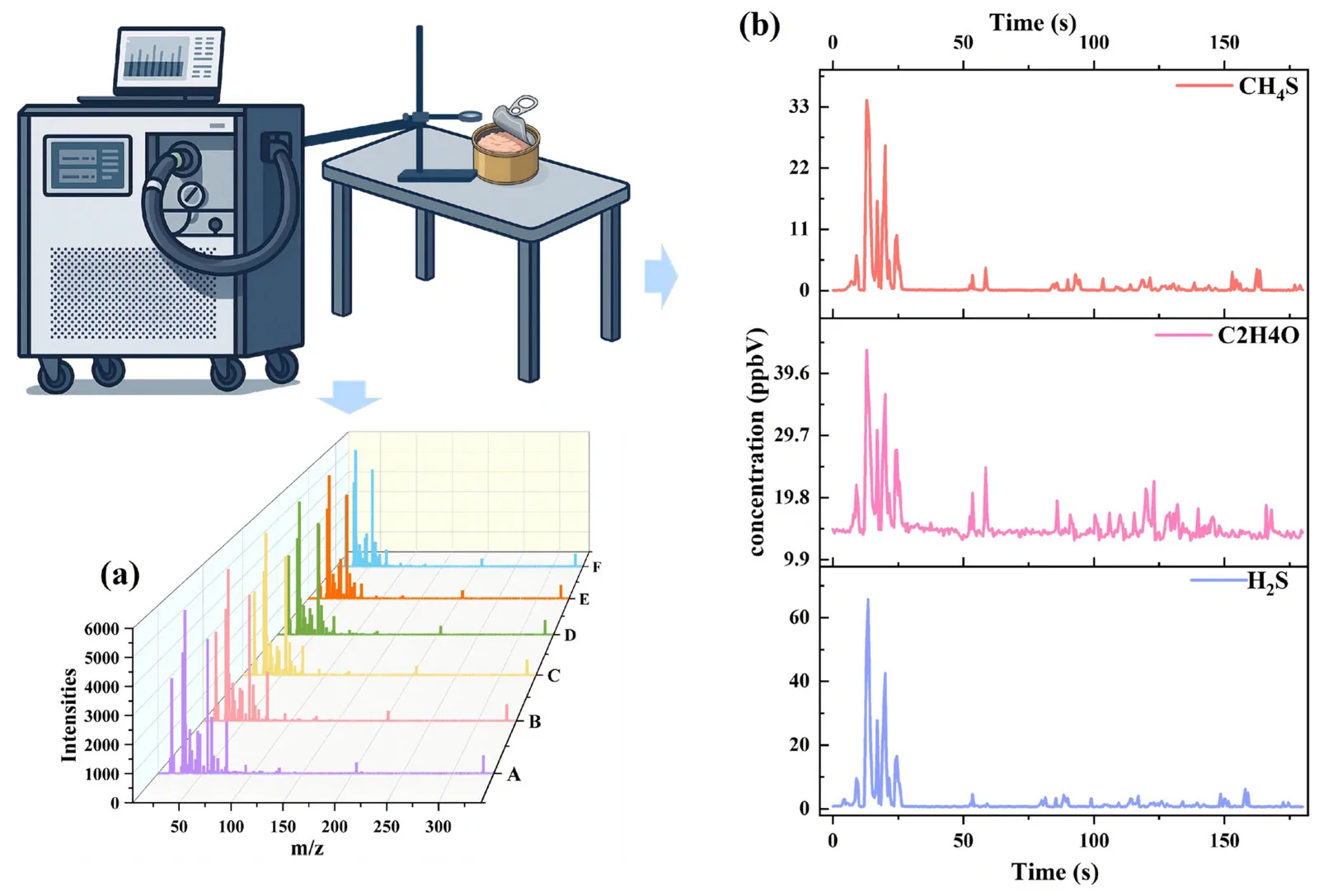
PTR-ToF-MS fingerprints and representative time-resolved responses during can opening. (**a**) Three-dimensional mass-spectral fingerprints of Groups A–F over *m*/*z* 20–350. (**b**) Representative time courses of features at *m*/*z* 49.011, 45.033, and 34.995. The opening moment was defined as t = 0 s. The −5 to 0 s interval represents the pre-opening baseline window, 0–60 s the burst phase, and 60–180 s the post-burst phase. Signals were background-corrected using the corresponding can-specific pre-opening baseline and are reported as semi-quantitative ppbv estimates.

PTR-ToF-MS monitoring showed a rapid change in the volatile signal immediately after opening. Three-dimensional fingerprints recorded from 0 to 180 s revealed strong early responses for a number of low-*m*/*z* features, followed by a marked decline in signal intensity (Figure 2a). Representative time profiles for *m*/*z* 49.011, 45.033, and 34.995 further illustrate this behavior (Figure 2b). For these features, the response increased rapidly after t = 0 s and then decreased toward lower levels as the post-opening period progressed. This burst–decay pattern is consistent with the rapid dispersion of volatiles after a closed package is opened [21]. The strongest changes were concentrated in the early part of the measurement period, supporting the use of 0–60 s as the main burst phase for subsequent feature comparison. The −5 to 0 s interval was retained as the can-specific pre-opening baseline, while 60–180 s was treated as the post-burst phase. The complete time series was retained for kinetic interpretation rather than reducing the opening event to a single equilibrium headspace measurement.

### 3.2. Burst-Phase Feature Fingerprints and Between-Treatment Differences

**Figure 3:**
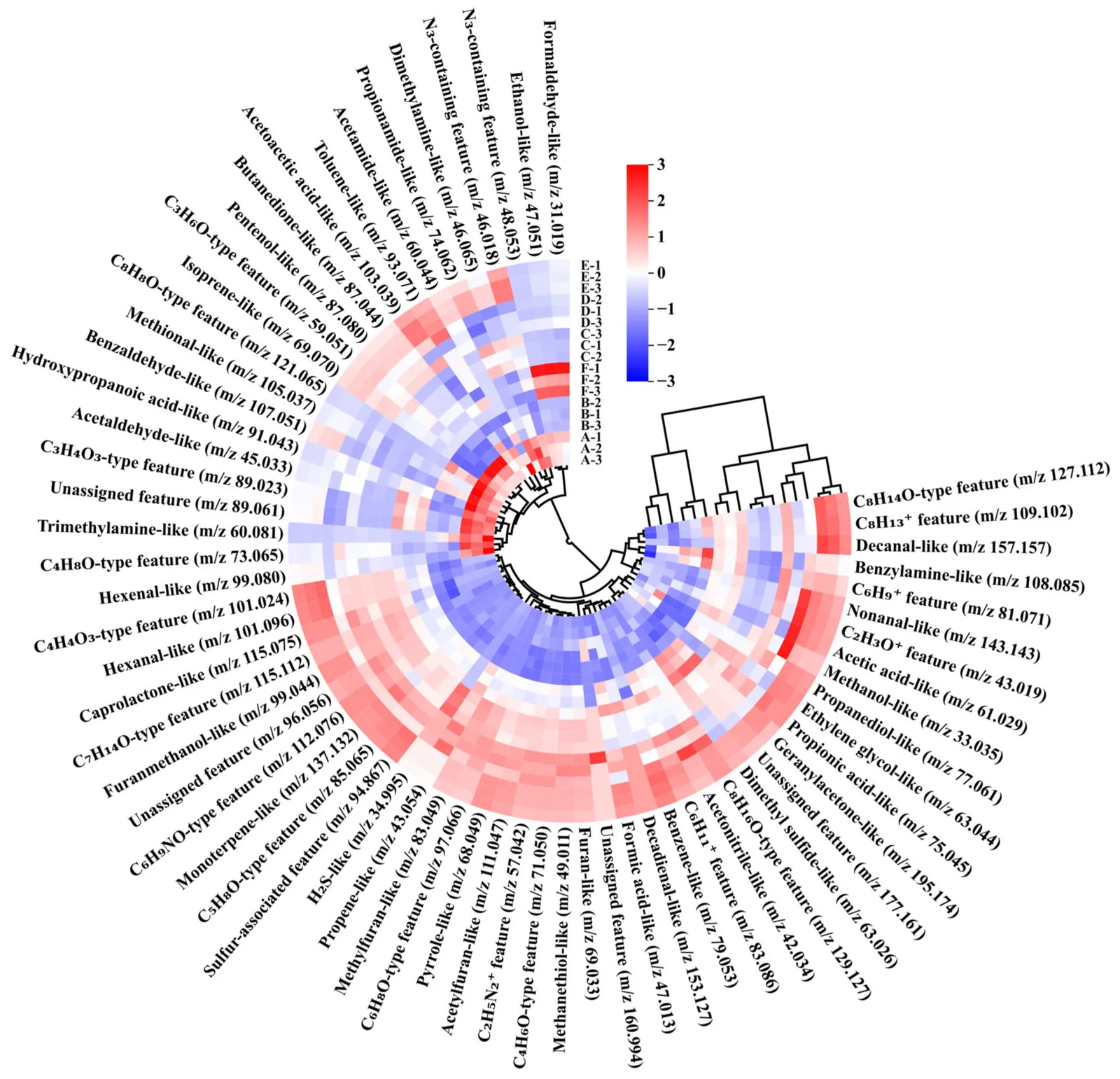
Hierarchical clustering heatmap of burst-phase PTR-ToF-MS feature responses in canned Antarctic krill. Feature responses were calculated as the mean background-corrected signal over 0–60 s (C_corr,0−60_) for each independent can and Z-score-standardized across samples before hierarchical clustering. Each column represents one independent can (*n* = 3 per treatment), and each row represents one retained *m*/*z* feature. The color scale shows relative standardized response levels and is intended for comparison of treatment-related patterns rather than absolute signal magnitudes among different features. Feature labels follow the putative “-like”, formula-/family-level, or unassigned-feature annotation scheme described in Section 2.3.

Volatile release after can-opening is a non-equilibrium process in which concentrated headspace constituents disperse into the surrounding air within seconds, producing a transient post-opening odor plume [22]. Conventional equilibrium or quasi-equilibrium headspace approaches such as HS-SPME/GC–MS are well suited to compositional profiling under defined sampling conditions, but the enrichment and sampling processes do not preserve the original seconds-scale peak-and-decay sequence of the opening event [8,9]. PTR-ToF-MS, by contrast, records rapid changes in volatile signals directly at sub-second to second time resolution [11]. Based on the time segmentation defined in Figure 2b, treatment differences were evaluated using the mean background-corrected response over 0–60 s (C_corr,0−60_). The complete group-level feature dataset is provided in Supplementary Table S1, and Figure 3 shows the Z-score-standardized hierarchical clustering heatmap of the burst-phase fingerprints.

The heatmap showed that pretreatment and retorting changed the burst-phase fingerprints in different ways rather than causing a uniform increase or decrease with processing severity. Group B generally showed lower relative responses than Group A for several low-*m*/*z* features, indicating a marked change after soaking and short-time blanching. Retorting produced a different pattern, with increases in several sulfur-associated and oxygenated features. The four F_0_-equivalent retorted groups also retained distinct fingerprints. Several sulfur-associated features were relatively prominent in Groups C–E, whereas Group F showed stronger responses for selected low-molecular oxygenated features. Thus, similar accumulated lethality did not produce identical post-opening volatile patterns, and the temperature–time pathway remained influential. At the chemical-family level, the prominence of oxygenated and sulfur-/nitrogen-associated responses was broadly consistent with volatile classes previously reported for Antarctic krill using GC-based approaches [5,6,7]. However, direct numerical comparison is not appropriate because the present PTR-ToF-MS data represent semi-quantitative time-resolved feature responses, whereas the GC-based studies characterize chromatographically separated compounds under different sampling conditions.

Acetaldehyde-like (*m*/*z* 45.033) showed a clear processing-related change. Its mean burst-phase response was relatively high in raw krill (Group A, 16.2 ppbv) but decreased markedly after soaking and blanching (Group B, 1.69 ppbv). After retorting, the response increased from 4.10 ppbv in Group C to 11.0 ppbv in Group F, although it remained below the level observed in Group A. The decrease from Group A to Group B may be related to the loss or redistribution of readily released, water-compatible constituents during soaking and blanching, together with changes in matrix–headspace partitioning [23,24,25]. The subsequent increase across the retorted groups is consistent with the additional formation of low-molecular carbonyl-related products during thermal processing and lipid oxidation [26]. The PTR-ToF-MS response itself does not establish the sensory contribution of this feature.

Ethanol-like (*m*/*z* 47.051) showed a different, non-monotonic pattern. Its mean response decreased from 6.09 ppbv in Group A to 0.32 ppbv in Group B and remained relatively low in Groups C–E (0.85–2.21 ppbv), but increased sharply to 12.9 ppbv in Group F. Since all four retorted groups had similar F_0_ values, the high response in Group F cannot be explained by accumulated lethality alone. It instead reflects a response specific to the 121 °C high-temperature/short-time pathway, in which volatile formation, matrix release, and further thermal transformation may have occurred in a different balance. Thermal history can alter both the formation and subsequent transformation of volatile products during food processing [27].

Sulfur-associated features followed another pattern. H_2_S-like (*m*/*z* 34.995) increased from 0.95 and 0.04 ppbv in Groups A and B, respectively, to 22.4, 16.2, 10.0, and 3.14 ppbv in Groups C–F. Methanethiol-like (*m*/*z* 49.011) was also low in Groups A and B (1.64 and 0.02 ppbv, respectively) but increased after retorting, reaching 5.26–6.48 ppbv in Groups C–E before decreasing to 3.13 ppbv in Group F. These changes are compatible with thermal transformation of sulfur-containing amino acids and related precursors [28,29]. The decline toward Group F also shows that these sulfur-associated responses did not increase simply with retort temperature.

Several nitrogen-associated low-*m*/*z* features were more prominent in raw krill and decreased after pretreatment and retorting. Trimethylamine-like (*m*/*z* 60.081) decreased from 0.66 ppbv in Group A to 0.21 ppbv in Group B and to 0.01–0.05 ppbv in Groups C–F. The decrease after soaking and blanching is consistent with loss or redistribution of water-soluble low-molecular amines and related precursors [30], while subsequent heating can further alter amino-acid- and amine-related volatile chemistry [31].

Taken together, the burst-phase fingerprints show that soaking/blanching and retorting affected different feature families in different directions. More importantly, the four F_0_-equivalent thermal treatments retained distinguishable post-opening patterns, showing that the specific temperature–time history remained relevant even when accumulated sterilization lethality was similar. Chemical names in this section are used as putative feature annotations and not as definitive structural identifications.

### 3.3. Exploratory Multivariate Pattern Recognition and Screening of Burst-Phase Features

**Figure 4:**
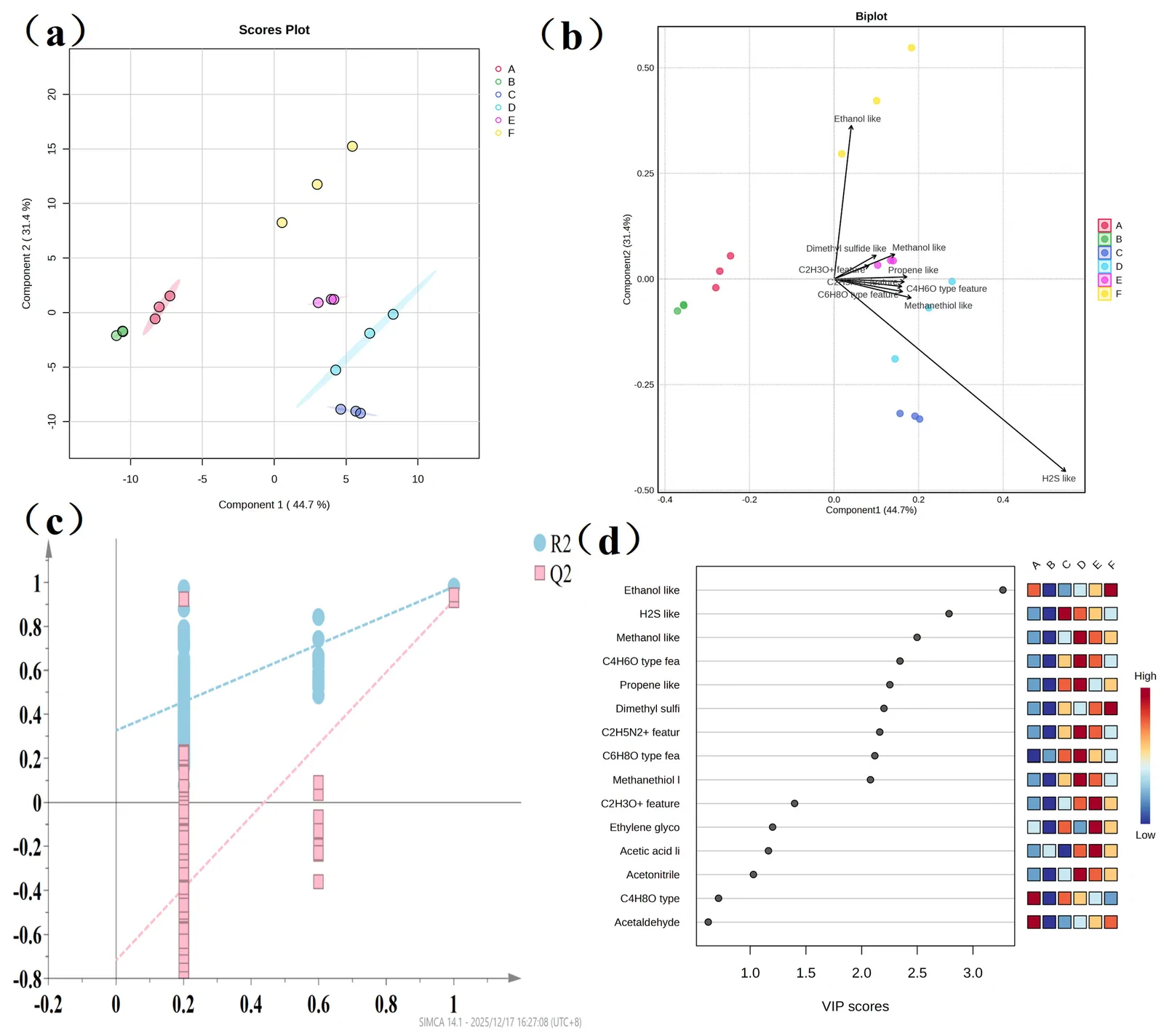
Exploratory PLS-DA of burst-phase (0–60 s) PTR-ToF-MS fingerprints and discriminant features. (**a**) PLS-DA scores plot for Groups A–F; each point represents one independent can (*n* = 3 per treatment). (**b**) Biplot showing sample scores and loading directions of representative features. (**c**) 200-iteration permutation test of the PLS-DA model (R^2^X(cum) = 0.937, R^2^Y(cum) = 0.953, Q^2^(cum) = 0.814). The dashed lines represent the regression lines for the permuted R^2^ and Q^2^ values. (**d**) VIP ranking and Z-score-standardized response patterns of features with VIP > 1. Feature names follow the putative “-like” or formula-/family-level annotation scheme described in Section 2.3. PLS-DA was used for exploratory pattern recognition and feature screening rather than predictive classification.

Multivariate chemometric approaches are commonly used to examine treatment-related patterns in complex volatile datasets [23,32]. Partial least squares–discriminant analysis (PLS-DA) was used here to examine differences in the burst-phase feature patterns among treatments (Figure 4). Each point in the score plot represents one independent can (*n* = 3 per treatment). The model gave R^2^X(cum) = 0.937, R^2^Y(cum) = 0.953, and Q^2^(cum) = 0.814. In the 200-iteration permutation test (Figure 4c), the permuted Q^2^ values were lower than that of the original model, and the Q^2^ regression line had a negative intercept at x = 0. These results support the use of the model for exploratory pattern recognition and feature screening within the present dataset [32]. The model was not used for predictive classification.

The first two latent variables accounted for 44.7% and 31.4% of the modeled variation, respectively, giving a cumulative value of 76.1% (Figure 4a). Groups A and B were mainly located on the negative side of Component 1, whereas the retorted groups (C–F) were located predominantly on the positive side. The transition from non-retorted to retorted samples therefore represented the main source of variation in the burst-phase fingerprint. Among the F_0_-equivalent retorted treatments, Group F showed a distinct displacement along Component 2 relative to Groups C–E, indicating that the 121 °C high-temperature/short-time treatment retained a different post-opening feature pattern despite a similar accumulated lethality.

The biplot (Figure 4b) showed that the position of Group F was associated mainly with ethanol-like (*m*/*z* 47.051) and formaldehyde-like (*m*/*z* 31.019). Both features showed relatively strong responses in Group F, consistent with the feature patterns described in Section 3.2. Thermal history can influence the formation and further transformation of low-molecular volatile products during food processing [33]. The loading directions shown in the biplot therefore indicate their contribution to multivariate separation rather than a specific formation pathway.

VIP scores were then used to identify features contributing strongly to treatment separation. Thirteen features had VIP > 1 and were retained for feature screening (Figure 4d). Several sulfur-associated features, including H_2_S-like (*m*/*z* 34.995), methanethiol-like (*m*/*z* 49.011), and dimethyl sulfide-like (*m*/*z* 63.026), showed low relative responses in Groups A and B and higher responses after retorting. Thermal reactions involving sulfur-containing precursors can generate volatile sulfur products during processing [28]. In contrast, ethanol-like (*m*/*z* 47.051) and formaldehyde-like (*m*/*z* 31.019) were relatively prominent in Group F. Processing-dependent changes in volatile formation and release can also arise from thermal and lipid-related transformations [34]. Other oxygenated features, including the C_4_H_6_O-type feature (*m*/*z* 71.050) and C_6_H_8_O-type feature (*m*/*z* 97.066), also varied among the F_0_-equivalent retorted treatments. Because Figure 4d is based on Z-score-standardized data, the color pattern reflects relative changes among treatments rather than absolute differences in response magnitude among chemical species.

**Figure 5:**
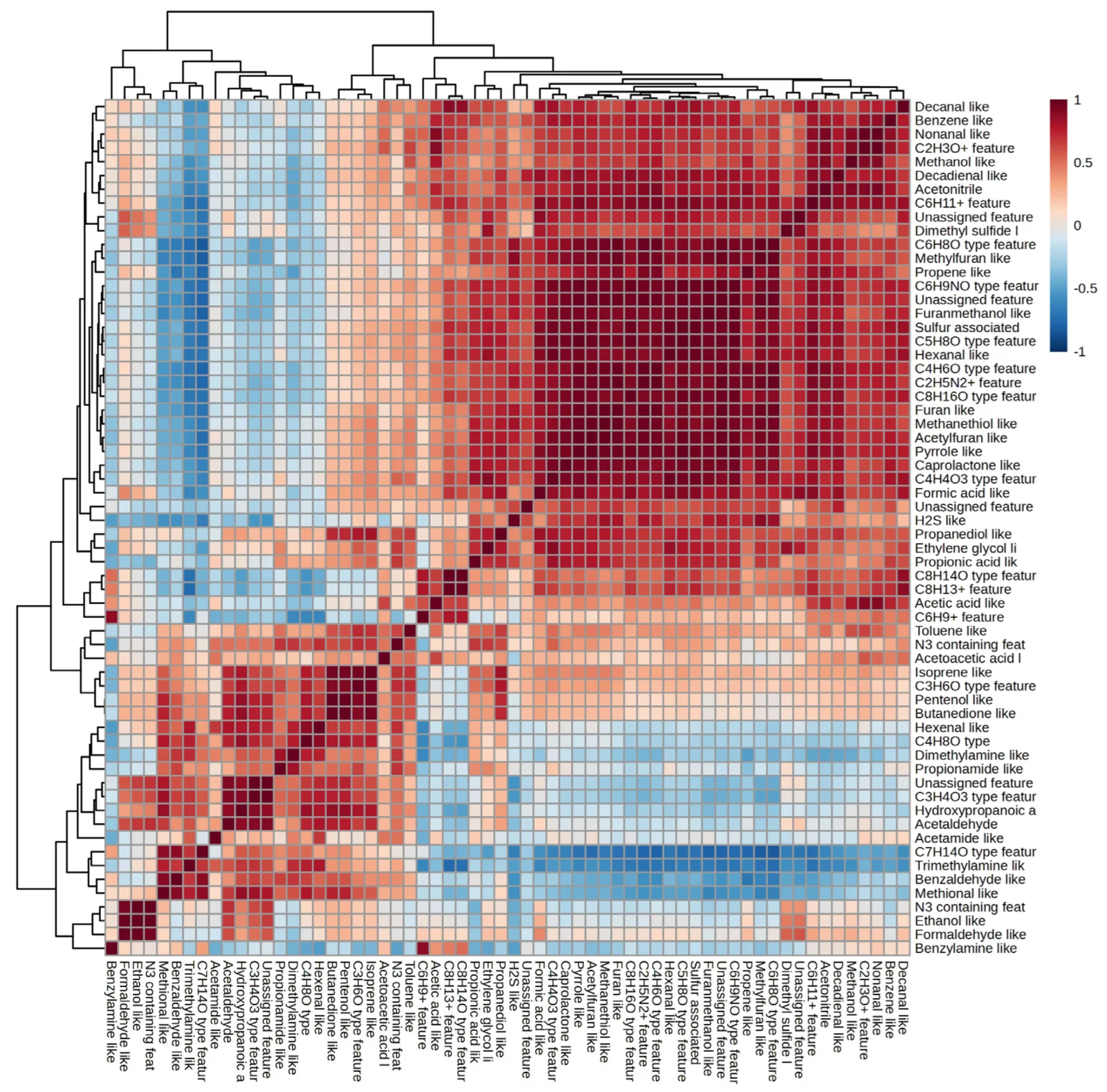
Pearson correlation heatmap of burst-phase (0–60 s) PTR-ToF-MS features. Pearson correlation coefficients were calculated across the 18 independent cans using the mean background-corrected response over 0–60 s (C_corr,0−60_) for each feature, followed by hierarchical clustering of the correlation matrix. Feature labels follow the putative “-like”, formula-/family-level, or unassigned-feature annotation scheme described in Section 2.3. The color scale represents Pearson correlation coefficients from −1 to 1. Correlations describe sample-level co-variation and do not indicate causal relationships or common formation pathways.

Pearson correlation analysis was used to examine sample-level co-variation among burst-phase features (Figure 5). Correlation coefficients were calculated across the 18 independent cans using C_corr,0−60_ for each feature. Several sulfur-associated features showed positive co-variation, including H_2_S-like (*m*/*z* 34.995), methanethiol-like (*m*/*z* 49.011), and dimethyl sulfide-like (*m*/*z* 63.026). Thermal transformation of sulfur-containing precursors provides a chemical context for the processing-dependent changes in these sulfur-associated responses [35], although the correlations themselves describe co-variation rather than common formation pathways. Among the oxygenated and carbonyl-related features, hexanal-like (*m*/*z* 101.096), the C_7_H_14_O-type feature (*m*/*z* 115.112), and the C_8_H_14_O-type feature (*m*/*z* 127.112) showed coordinated variation across the samples. Such co-variation is consistent with the broader formation and transformation of oxygenated volatile products during thermal and oxidative processes [27], but does not establish a shared precursor or causal relationship.

The PLS-DA, VIP, and correlation analyses consistently showed that pretreatment and retorting changed the burst-phase feature pattern, while the four F_0_-equivalent retort schedules remained distinguishable. The discriminant features identified here were further examined using the time-domain descriptors C_max,0–60_ and AUC_0–60_.

### 3.4. Time-Domain Characteristics of Dominant Burst-Phase Features During Can Opening

#### 3.4.1. Overall Burst-Phase Release Characteristics and Feature-Response Contribution

Twenty-five features met the time-domain screening criteria defined in Section 2.4 and were used to characterize the 0–60 s burst phase. For each feature, C_max,0–60_ describes the strongest background-corrected response after opening, whereas AUC_0–60_ represents the cumulative response over the complete burst interval.

**Figure 6:**
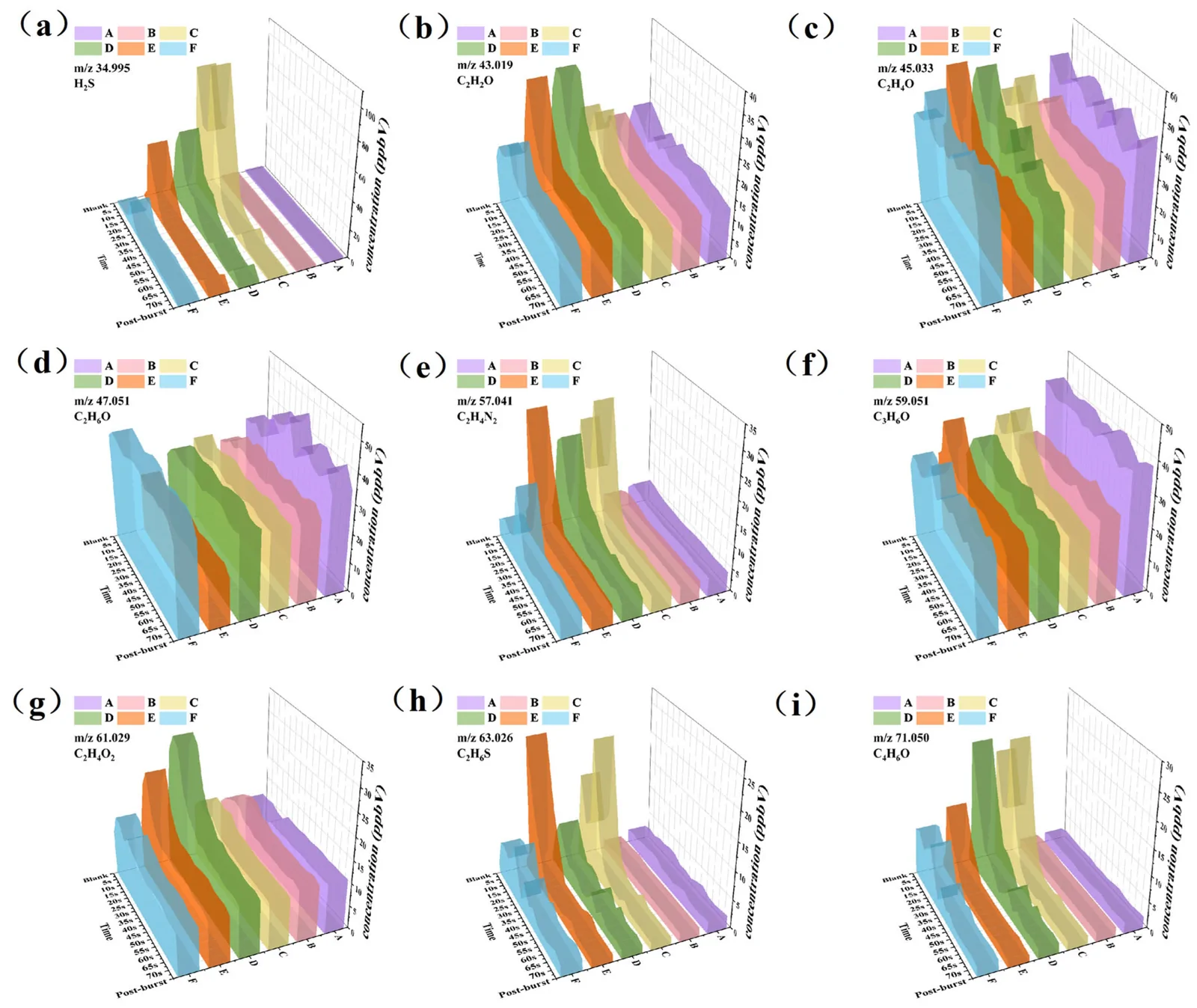
Time-resolved responses of the nine highest-ranked PTR-ToF-MS features during the 0–60 s burst phase after can-opening. (**a**–**i**) Mean time profiles of the selected features from three independent cans per treatment (*n* = 3). Signals were corrected using the corresponding can-specific −5 to 0 s pre-opening baseline and are expressed as semi-quantitative ppbv responses. The features are: (**a**) H_2_S-like (*m*/*z* 34.995); (**b**) C_2_H_3_O^+^ feature (*m*/*z* 43.019); (**c**) acetaldehyde-like (*m*/*z* 45.033); (**d**) ethanol-like (*m*/*z* 47.051); (**e**) C_2_H_5_N_2_^+^ feature (*m*/*z* 57.042); (**f**) C_3_H_6_O-type feature (*m*/*z* 59.051); (**g**) acetic acid-like (*m*/*z* 61.029); (**h**) dimethyl sulfide-like (*m*/*z* 63.026); and (**i**) C_4_H_6_O-type feature (*m*/*z* 71.050). The profiles are intended for treatment-wise comparison of the same *m*/*z* feature rather than direct quantitative comparison among different features.

The time profiles showed that the opening response was concentrated in the first few seconds. Twenty-three of the twenty-five features reached their maximum responses within the first 10 s, including two that peaked within the first 5 s. Most signals then declined rapidly or approached lower response levels. Similar burst–decay behavior has been reported in real-time PTR-MS studies of food aroma release [11]. The first 5–10 s therefore contained the most pronounced changes in the measured post-opening volatile response. The time profiles of the nine highest-ranked features are shown in Figure 6.

Although most features shared a rapid rise followed by decay, their peak magnitude, peak timing, and subsequent response trajectories differed. C_max,0–60_ and AUC_0–60_ captured different aspects of this behavior. C_max,0–60_ reflected the magnitude of the strongest transient response, whereas AUC_0–60_ described the cumulative response throughout the first 60 s after opening.

AUC_0–60_ also differed substantially among the 25 features (Table 1). Based on the mean AUC_0–60_ response, the nine highest-ranked features accounted for 76.78% of the cumulative response within the retained candidate set. Acetaldehyde-like (*m*/*z* 45.033), H_2_S-like (*m*/*z* 34.995), and ethanol-like (*m*/*z* 47.051) made the largest contributions, accounting for 17.3%, 15.8%, and 11.2%, respectively. Other highly ranked signals included the C_2_H_3_O^+^ feature (*m*/*z* 43.019), C_3_H_6_O-type feature (*m*/*z* 59.051), C_2_H_5_N_2_^+^ feature (*m*/*z* 57.042), C_4_H_6_O-type feature (*m*/*z* 71.050), acetic acid-like (*m*/*z* 61.029), and dimethyl sulfide-like (*m*/*z* 63.026). The AUC_0–60_ ranking reflects the relative PTR-ToF-MS response of each feature within the retained candidate set and does not represent absolute chemical abundance or odor activity. Instrumental response alone should not be equated directly with sensory relevance [36].

#### 3.4.2. Putative Chemical-Family Composition of the Nine Highest-Ranked Features

Based on measured *m*/*z*, plausible ion formulae, PTR ion chemistry, and literature information, the nine highest-ranked features were interpreted mainly at the chemical-family level. Oxygenated features accounted for most of the selected signals, accompanied by sulfur- and nitrogen-associated features.

The oxygenated group included acetaldehyde-like (*m*/*z* 45.033), ethanol-like (*m*/*z* 47.051), the C_2_H_3_O^+^ feature (*m*/*z* 43.019), C_3_H_6_O-type feature (*m*/*z* 59.051), acetic acid-like (*m*/*z* 61.029), and the C_4_H_6_O-type feature (*m*/*z* 71.050). These signals represent a mixed group of low-molecular oxygen-containing features related broadly to carbonyl, alcohol, organic-acid, and unsaturated oxygenated chemistry. Thermal processing can promote Maillard-related reactions, lipid oxidation, and other heat-induced transformations that generate or modify low-molecular oxygenated products [37]. Carbonyl and dicarbonyl intermediates can also react with food proteins during heating, adding further complexity to the thermal chemistry of oxygenated compounds in food matrices [38]. The predominance of oxygenated features among the nine highest-ranked signals shows that this chemical family formed a major part of the measured opening-burst response.

Two of the highly ranked features were sulfur-associated: H_2_S-like (*m*/*z* 34.995) and dimethyl sulfide-like (*m*/*z* 63.026). Both increased markedly after retorting, in agreement with the processing trends described in Section 3.2 and Section 3.3. Heating can alter sulfur-containing amino acids and other sulfur precursors, leading to changes in volatile sulfur products [39]. This provides a reasonable chemical background for the stronger sulfur-associated responses observed after retorting.

The remaining highly ranked nitrogen-associated signal was the C_2_H_5_N_2_^+^ feature (*m*/*z* 57.042). Its relatively high AUC_0–60_ indicates that nitrogen-containing responses also contributed to the opening-burst fingerprint. Thermal reactions involving amino compounds, including Maillard-related chemistry, can generate a range of nitrogen-containing volatile products [40]. However, the measured *m*/*z* alone does not support assignment of this feature to a single nitrogen-containing compound.

The nine highest-ranked features therefore consisted mainly of low-molecular oxygenated responses, together with prominent sulfur- and nitrogen-associated signals. These assignments are used at the feature or chemical-family level and do not represent definitive structural identification.

#### 3.4.3. Kinetic Differences Among Representative Burst-Phase Features

The representative burst-phase features differed in both pre-opening baseline levels and opening-induced response magnitudes. Acetaldehyde-like (*m*/*z* 45.033) and ethanol-like (*m*/*z* 47.051) showed relatively high mean pre-opening baselines of 32.4 and 29.7 ppbv, respectively. After opening, their mean baseline-corrected C_max,0–60_ values reached 30.5 and 21.0 ppbv. Their high AUC_0–60_ values therefore reflected not only strong early responses but also sustained signals during the 0–60 s burst phase.

Several other low-molecular oxygenated features also responded rapidly after opening. The C_2_H_3_O^+^ feature (*m*/*z* 43.019) and C_3_H_6_O-type feature (*m*/*z* 59.051) reached mean baseline-corrected C_max,0–60_ values of 16.0 and 14.9 ppbv, respectively, while acetic acid-like (*m*/*z* 61.029) reached 9.84 ppbv. These features contributed strongly to the early post-opening response despite differences in their individual time profiles.

A different pattern was observed for several sulfur- and nitrogen-associated features. H_2_S-like (*m*/*z* 34.995) had a low mean pre-opening baseline of 1.28 ppbv but showed the largest mean baseline-corrected C_max,0–60_ among the retained features, reaching 36.6 ppbv. Dimethyl sulfide-like (*m*/*z* 63.026) increased from a mean baseline of 3.25 ppbv to a baseline-corrected C_max,0–60_ of 9.60 ppbv. The C_2_H_5_N_2_^+^ feature (*m*/*z* 57.042) also showed a pronounced opening response, with a baseline-corrected C_max,0–60_ of 14.5 ppbv. These signals were characterized by relatively low pre-opening backgrounds followed by rapid increases after the can was opened.

The C_4_H_6_O-type feature (*m*/*z* 71.050) showed a similar release pattern. Its mean pre-opening baseline was 1.83 ppbv, whereas its baseline-corrected C_max,0–60_ reached 12.2 ppbv. This feature therefore provides another example of a low-background signal that became prominent during the opening burst.

The selected features thus showed distinct release kinetics. Some were characterized by relatively high background levels together with sustained post-opening responses, whereas others showed low pre-opening signals followed by sharp opening-induced increases. These differences show why the full time-resolved profiles are useful for describing the opening event and cannot be replaced by a single headspace value.

#### 3.4.4. Integrated Interpretation of Opening-Burst Release Patterns

The time-resolved profiles revealed two broad response patterns during the 0–60 s burst phase. Several low-molecular oxygenated features showed appreciable pre-opening backgrounds together with sustained responses after opening, whereas several sulfur- and nitrogen-associated features showed relatively low baseline signals followed by pronounced opening-induced increases. Twenty-three of the twenty-five retained features reached their maximum responses within the first 10 s, including two that peaked within the first 5 s. The opening event was therefore dominated by rapid changes occurring within the first few seconds.

The four retort treatments also retained distinguishable burst-phase fingerprints despite having similar F_0_ values. Differences were particularly evident in sulfur-associated and low-molecular oxygenated features. Similar accumulated sterilization lethality therefore did not result in identical post-opening volatile behavior. The specific temperature–time history affected both the relative feature pattern and the temporal course of the volatile burst released after opening.

These results show that the opening event itself carries information that is not apparent from a single static headspace value. Time-resolved monitoring can therefore be used to characterize the short-lived post-opening volatile fingerprint and to compare how different thermal processing routes shape this initial release event. The sensory relevance of individual features remains to be established by direct sensory evaluation and compound-level identification.

## 4. Conclusions

This study characterized the transient volatile release immediately after opening canned Antarctic krill using time-resolved PTR-ToF-MS under F_0_-equivalent processing conditions. Twenty-three of the 25 retained features reached their maximum responses within 10 s, including two within 5 s, confirming that the earliest post-opening period contained the most pronounced volatile changes. The nine highest-ranked features accounted for 76.78% of the cumulative AUC_0–60_ response within the retained candidate feature set. Despite similar F_0_ values, the four retort treatments produced distinguishable opening-burst fingerprints. Sulfur-associated features varied markedly among thermal treatments, while several low-molecular oxygenated features were particularly prominent after the 121 °C high-temperature/short-time treatment. These results indicate that equivalent sterilization lethality does not necessarily produce equivalent post-opening volatile behavior, and that the specific temperature–time history remains important.

Time-resolved PTR-ToF-MS therefore provides a direct way to characterize the short-lived volatile environment encountered immediately after opening and to compare processing-dependent release patterns in retort-canned foods. Further chromatographic identification and sensory evaluation will help clarify the contribution of individual transient features to the aroma perceived at opening.

## Figures and Tables

**Table 1:** Top 25 burst-phase (0–60 s) PTR-ToF-MS features ranked by AUC_0–60_ contribution.

Rank	Measured *m*/*z*	Putative Formula/Feature Annotation	C− Base (−5–0 s), Mean (ppbv)	C_max,0–60_, Raw, Mean (ppbv)	C_max,0–60_, Baseline-Corrected, Mean (ppbv)	AUC_0–60_, Mean (ppbv·s)	Contribution (%)
1	45.033	C_2_H_4_O Acetaldehyde-like	32.4	62.9	30.5	625	17.31
2	34.995	H_2_S H_2_S-like	1.28	37.9	36.6	570	15.78
3	47.051	C_2_H_6_O Ethanol-like	29.7	50.7	21	405	11.20
4	43.019	C_2_H_2_O C_2_H_3_O^+^ feature	13	29.1	16	276	7.63
5	59.051	C_3_H_6_O C_3_H_6_O-type feature	27.8	42.7	14.9	248	6.87
6	57.042	C_2_H_4_N_2_ C_2_H_5_N_2_^+^ feature	5.06	19.6	14.5	197	5.44
7	71.050	C_4_H_6_O C_4_H_6_O-type feature	1.83	14.1	12.2	178	4.93
8	61.029	C_2_H_4_O_2_ Acetic acid-like	12.2	22	9.84	143	3.97
9	63.026	C_2_H_6_S Dimethyl sulfide-like	3.25	12.9	9.6	132	3.65
10	49.011	CH_4_S Methanethiol-like	0.69	10.7	10	129	3.57
11	97.066	C_6_H_8_O C_6_H_8_O-type feature	3.7	11.1	7.37	127	3.51
12	69.070	C_5_H_8_ Isoprene-like	3.38	11.2	7.86	121	3.34
13	108.085	C_7_H_9_N Benzylamine-like	4.1	10.1	6.02	95.4	2.64
14	77.061	C_3_H_8_O_2_ Propanediol-like	3.31	10.5	7.23	91.7	2.54
15	81.071	C_6_H_8_ C_6_H_9_^+^ feature	3.25	8.85	5.6	90.3	2.50
16	87.044	C_4_H_6_O_2_ Butanedione-like	3.48	6.91	3.43	64.3	1.78
17	121.065	C_8_H_8_O C_8_H_8_O-type feature	4.31	6.39	2.08	27.2	0.75
18	99.044	C_5_H_6_O_2_ Furanmethanol-like	3.63	5.82	2.2	24.2	0.67
19	85.065	C_5_H_8_O C_5_H_8_O-type feature	2.91	4.78	1.87	23.7	0.66
20	89.061	C_5_H_8_O Unassigned feature	3.49	4.27	0.78	10.6	0.29
21	157.157	C_10_H_20_O Decanal-like	5.03	5.39	0.36	9.62	0.27
22	99.080	C_6_H_10_O Hexenal-like	4.34	4.92	0.58	8.96	0.25
23	94.867	C_2_H_6_S_2_ Sulfur-associated feature	3.52	4.04	0.52	7.69	0.21
24	143.143	C_9_H_18_O Nonanal-like	4.45	4.77	0.32	5.23	0.15
25	60.081	C_3_H_9_N Trimethylamine-like	1.89	1.96	0.08	3.46	0.10

Note: Values are arithmetic means of three independent cans (*n* = 3) and are semi-quantitative PTR-ToF-MS responses. C_max,0–60_ and AUC_0–60_ were calculated as defined in Section 2.4. Putative formulas are software-assisted assignments from the PTR-ToF-MS data-processing workflow, and feature annotations do not represent definitive structural identifications. Contribution (%) represents the relative AUC_0–60_ feature-response contribution within the retained candidate feature set and should not be interpreted as absolute chemical abundance or sensory contribution.

## Data Availability

The original contributions presented in this study are included in the article/Supplementary Materials. Further inquiries can be directed to the corresponding author.

## Supplementary Materials

The following supporting information can be downloaded at https://www.mdpi.com/article/10.3390/foods15172968/s1, Table S1: Putative feature annotations and burst-phase (0–60 s) mean background-corrected PTR-ToF-MS responses of canned Antarctic krill under different processing routes.

## References

[1] Sun P., Lin J., Ren X., Zhang B., Liu J., Zhao Y., Li D. (2022). Effect of heating on protein denaturation, water state, microstructure, and textural properties of Antarctic krill (*Euphausia superba*) meat. Food Bioprocess Technol..

[2] Sun P., Zhang X., Ren X., Cao Z., Zhao Y., Man H., Li D. (2023). Effect of basic amino acid pretreatment on the quality of canned Antarctic krill. Food Bioprocess Technol..

[3] Tamanna N., Mahmood N. (2015). Food processing and Maillard reaction products: Effect on human health and nutrition. Int. J. Food Sci..

[4] Gonzalez-Estanol K., Pedrotti M., Fontova-Cerdà M., Khomenko I., Biasioli F., Stieger M. (2024). Influence of chewing rate and food composition on in vivo aroma release and perception of composite foods. J. Agric. Food Chem..

[5] Fan Y., Li Z., Xue Y., Hou H., Xue C. (2017). Identification of volatile compounds in Antarctic krill (*Euphausia superba*) using headspace solid-phase microextraction and GC–MS. Int. J. Food Prop..

[6] Sun P., Lin S., Li X., Li D. (2024). Different stages of flavor variations among canned Antarctic krill (*Euphausia superba*)*:* Based on GC–IMS and PLS–DA. Food Chem..

[7] Sun P., Lin S., Li X., Li D. (2025). Effects of sterilization intensity on the flavor profile of canned Antarctic krill (*Euphausia superba*): Moderate vs. excessive. Food Chem..

[8] Bicchi C., Cordero C., Liberto E., Sgorbini B., Rubiolo P. (2008). Headspace sampling of the volatile fraction of vegetable matrices. J. Chromatogr. A.

[9] Reyes-Garcés N., Gionfriddo E., Gómez-Ríos G.A., Alam M.N., Boyacı E., Bojko B., Singh V., Grandy J., Pawliszyn J. (2018). Advances in solid-phase microextraction and perspective on future directions. Anal. Chem..

[10] Blake R.S., Monks P.S., Ellis A.M. (2009). Proton-transfer reaction mass spectrometry. Chem. Rev..

[11] Majchrzak T., Wojnowski W., Wasik A. (2021). Revealing dynamic changes of the volatile profile of food samples using PTR–MS. Food Chem..

[12] Muñoz-González C., Canon F., Feron G., Guichard E., Pozo-Bayón M.A. (2019). Assessment of wine aroma persistence using an in vivo PTR-ToF-MS approach and its relationship with salivary parameters. Molecules.

[13] Gonzalez-Estanol K., Khomenko I., Cliceri D., Biasioli F., Stieger M. (2023). In vivo aroma release and perception of composite foods using nose-space PTR–ToF–MS analysis with temporal check-all-that-apply. Food Res. Int..

[14] Wieland F., Gloess A.N., Keller M., Wetzel A., Schenker S., Yeretzian C. (2012). Online monitoring of coffee roasting by proton-transfer-reaction time-of-flight mass spectrometry (PTR-ToF-MS): Towards real-time process control for a consistent roast profile. Anal. Bioanal. Chem..

[15] Zanin R.C., Smrke S., Kurozawa L.E., Yamashita F., Yeretzian C. (2020). Novel experimental approach to study aroma release upon reconstitution of instant coffee products. Food Chem..

[16] Anderson N., Larkin J., Cole M., Skinner G., Whiting R., Gorris L., Rodriguez A., Buchanan R., Stewart C., Hanlin J. (2011). Food safety objective approach for controlling Clostridium botulinum growth and toxin production in commercially sterile foods. J. Food Prot..

[17] Ni Q., Khomenko I., Gallo L., Biasioli F., Bittante G. (2020). Rapid profiling of the volatilome of cooked meat by PTR-ToF-MS: Characterization of chicken, turkey, pork, veal, and beef meat. Foods.

[18] Ghanbari J., Khajoei-Nejad G., Erasmus S.W., van Ruth S.M. (2019). Identification and characterisation of volatile fingerprints of saffron stigmas and petals using PTR-ToF-MS: Influence of nutritional treatments and corm provenance. Ind. Crops Prod..

[19] Benozzi E., Romano A., Capozzi V., Makhoul S., Cappellin L., Khomenko I., Aprea E., Scampicchio M., Spano G., Märk T.D. (2015). Monitoring lactic fermentation driven by different starter cultures via direct-injection mass spectrometric analysis of flavour-related volatile compounds. Food Res. Int..

[20] Aubourg S.P. (2001). Loss of quality during the manufacture of canned fish products: A review. Food Sci. Technol. Int..

[21] Mateus M.-L., Lindinger C., Gumy J.-C., Liardon R. (2007). Release kinetics of volatile organic compounds from roasted and ground coffee: Online measurements by PTR-MS and mathematical modeling. J. Agric. Food Chem..

[22] Taylor A.J., Beauchamp J.D., Langford V.S. (2021). Non-destructive and high-throughput—APCI-MS, PTR-MS, and SIFT-MS as methods of choice for exploring flavor release. Dynamic Flavor: Capturing Aroma Using Real-Time Mass Spectrometry.

[23] Smolinska A., Pellacani S., Skawinski M., Durante C. (2025). On the road to automation: A comparative review of chemometric strategies for GC–MS data analysis. TrAC Trends Anal. Chem..

[24] Zardin E., Tyapkova O., Buettner A., Beauchamp J. (2014). Performance assessment of proton-transfer-reaction time-of-flight mass spectrometry (PTR-ToF-MS) for analysis of isobaric compounds in food-flavour applications. LWT Food Sci. Technol..

[25] Zhang L., Yu Y., Wen Q., Nie S., Hu Y., Tan C., Tu Z. (2025). Decoding the effects of brining time on the sensory quality, physicochemical properties, and flavor characteristics of marinated grass carp meat. Food Chem. X.

[26] Hu Y., Zhao G., Yin F., Liu Z., Wang J., Qin L., Zhou D., Shahidi F., Zhu B. (2022). Effects of roasting temperature and time on aldehyde formation derived from lipid oxidation in scallop (*Patinopecten yessoensis*) and the deterrent effect of bamboo-leaf antioxidants. Food Chem..

[27] Grebenteuch S., Kroh L.W., Drusch S., Rohn S. (2021). Formation of secondary and tertiary volatile compounds resulting from the lipid oxidation of rapeseed oil. Foods.

[28] Luo D., Tian B., Li J., Zhang W., Bi S., Fu B., Jing Y. (2024). Mechanisms underlying the formation of main volatile odor sulfur compounds in foods during thermal processing. Compr. Rev. Food Sci. Food Saf..

[29] Cerny C., Parker J.K., Elmore J.S., Methven L. (2015). The role of sulfur chemistry in thermal generation of aroma. Flavour Development, Analysis and Perception in Food and Beverages.

[30] Wu T., Wang M., Wang P., Tian H., Zhan P. (2022). Advances in the formation and control of undesirable flavors in fish. Foods.

[31] Ferreira I.M., Guido L.F. (2018). Impact of wort amino acids on beer flavour: A review. Fermentation.

[32] Stefanuto P.-H., Smolinska A., Focant J.-F. (2021). Advanced chemometric and data-handling tools for GC×GC-ToF-MS: Application of chemometrics and related advanced data handling in chemical separations. TrAC Trends Anal. Chem..

[33] Chu B., Zhang C., Song Y., Zhou H., Chen X., Gu Q. (2025). Effects of thermal sterilization conditions on flavor and lipid oxidation of sauced duck necks. Foods.

[34] Liu B., Mao Y., Wang C., Zhao Y., Liu K., Xu X., Yu F., Zhao Y. (2025). Effects of twin-screw extrusion conditions on the flavor quality of sturgeon mince: Lipid metabolic pathways. Food Chem..

[35] McGorrin R.J. (2011). The significance of volatile sulfur compounds in food flavors. Volatile Sulfur Compounds in Food.

[36] Starowicz M. (2021). Analysis of volatiles in food products. Separations.

[37] El Hosry L., Elias V., Chamoun V., Halawi M., Cayot P., Nehme A., Bou-Maroun E. (2025). Maillard reaction: Mechanism, influencing parameters, advantages, disadvantages, and food-industrial applications—A review. Foods.

[38] Bao Y., Gou H., Sánchez-Terrón G., Zhang L., Zamora R., Hidalgo F.J., Estévez M. (2025). Reactions between dicarbonyls and food proteins: When lipid oxidation and the Maillard reaction meet protein oxidation. Food Chem..

[39] Liu C., Lu L., Yang C., Niu C., Wang J., Zheng F., Li Q. (2022). Effects of thermal treatment on alliin and its related sulfides during black garlic processing. LWT.

[40] Shi B., Guo X., Liu H., Jiang K., Liu L., Yan N., Farag M., Liu L. (2024). Dissecting Maillard reaction production in fried foods: Formation mechanisms, sensory characteristic attribution, control strategy, and gut homeostasis regulation. Food Chem..

