# Supplementary material for: A Time-Resolved PTR-ToF-MS Workflow for Characterizing Opening-Burst Volatile Features in Canned Antarctic Krill Under Equivalent F0 Processing
Source: Foods. 2026 Aug 24;15(17):2968. doi: 10.3390/foods15172968 (PMC13565579; doi:10.3390/foods15172968)
Supplement: Supplementary file 1 [file foods-15-02968-s001.zip › foods-4482100-supplementary.pdf]

Table S1. Putative feature annotations and burst-phase (0–60 s) mean background-corrected PTR-ToF-MS responses of canned Antarctic krill under different processing routes.

Measured m/z	Putative formula	Putative annotation	Broad feature family	Group A (ppbv)	Group B (ppbv)	Group C (ppbv)	Group D (ppbv)	Group E (ppbv)	Group F (ppbv)
31.019	CH_2OH^+	Formaldehyde-like	Carbonyl-related	0.47	0.04	0.07	0.29	0.32	1.21
33.035	CH_4OH^+	Methanol-like	Alcohol-related	1.68	0.74	2.86	5.81	5.19	4.66
34.995	H_2SH^+	H_2S-like	Sulfur-associated	0.95	0.04	22.4	16.2	10.0	3.14
42.034	$C_2H_3NH^+$	Acetonitrile-like	Nitrogen-associated	0.26	0.03	1.06	2.44	1.98	1.39
43.019	$C_2H_2OH^+$	$C_2H_3O^+$ feature	Carbonyl-related	2.16	1.33	2.21	4.46	4.77	3.13
43.054	$C_3H_6H^+$	Propene-like	Hydrocarbon-related	1.69	0.72	5.07	6.85	4.21	4.88
45.033	$C_2H_4OH^+$	Acetaldehyde-like	Carbonyl-related	16.2	1.69	4.10	6.85	6.87	10.9
46.018	—	N_3-containing feature	Nitrogen-associated	0.37	0.04	0.23	0.14	0.42	0.15
46.065	$C_2H_7NH^+$	Dimethylamine-like	Nitrogen-associated	0.05	0.02	0.03	0.01	0.03	0.00
47.013	$CH_2O_2H^+$	Formic acid-like	Acid-related	0.12	0.03	0.25	0.35	0.49	0.38
47.051	$C_2H_6OH^+$	Ethanol-like	Alcohol-related	6.09	0.32	0.85	1.92	2.21	12.9
48.053	—	N_3-containing feature	Nitrogen-associated	0.24	0.01	0.03	0.05	0.03	0.45
49.011	CH_4SH^+	Methanethiol-like	Sulfur-associated	1.64	0.02	5.26	6.48	5.80	3.13
57.042	$C_2H_4N_2H^+$	$C_2H_5N_2^+$ feature	Nitrogen-associated	0.71	0.39	4.31	5.41	4.85	3.22
59.051	$C_3H_6OH^+$	C_3H_6O-type feature	Carbonyl-related	6.08	0.36	3.87	3.53	4.59	2.84
60.044	$C_2H_5NOH^+$	Acetamide-like	Amide-related	0.39	0.23	0.20	0.26	0.34	0.21
60.081	$C_3H_9NH^+$	Trimethylamine-like	Nitrogen-associated	0.66	0.21	0.05	0.03	0.01	0.01
61.029	$C_2H_4O_2H^+$	Acetic acid-like	Acid-related	1.07	1.53	0.70	2.96	3.72	2.22
63.026	$C_2H_6SH^+$	Dimethyl sulfide-like	Sulfur-associated	0.85	0.02	3.29	1.39	3.90	4.40
63.044	$C_2H_6O_2H^+$	Ethylene glycol-like	Oxygenated	2.13	0.10	3.75	1.90	4.52	2.85
68.049	$C_4H_5NH^+$	Pyrrole-like	N-heterocyclic	0.03	0.00	0.10	0.13	0.14	0.08
69.033	$C_4H_4OH^+$	Furan-like	Furan-related	0.19	0.06	0.62	0.75	0.66	0.45
69.070	$C_5H_8H^+$	Isoprene-like	Hydrocarbon/terpene-related	3.67	0.26	2.04	2.70	2.82	1.82
71.050	$C_4H_6OH^+$	C_4H_6O-type feature	Carbonyl-related	0.66	0.11	4.06	5.42	4.89	3.38
73.065	$C_4H_8OH^+$	C_4H_8O-type feature	Carbonyl-related	2.86	0.66	1.30	1.28	1.21	0.75
74.062	$C_3H_7NOH^+$	Propionamide-like	Amide-related	2.69	1.23	2.19	0.61	2.34	1.00
75.045	$C_3H_6O_2H^+$	Propionic acid-like	Acid-related	0.17	0.05	0.23	0.22	0.31	0.08
77.061	$C_3H_8O_2H^+$	Propanediol-like	Oxygenated	0.19	0.01	0.18	0.23	0.30	0.17
79.053	$C_6H_6H^+$	Benzene-like	Aromatic hydrocarbon-related	0.21	0.21	0.37	0.56	0.74	0.28
81.071	$C_6H_8H^+$	$C_6H_9^+$ feature	Hydrocarbon/terpene-related	0.75	1.61	0.84	1.55	1.65	1.46
83.049	$C_5H_6OH^+$	Methylfuran-like	Furan-related	0.06	0.04	1.17	1.09	1.02	0.63
83.086	$C_6H_{10}H^+$	$C_6H_{11}^+$ feature	Hydrocarbon-related	0.17	0.14	0.38	0.56	0.61	0.50

Measured m/z	Putative formula	Putative annotation	Broad feature family	Group A (ppbv)	Group B (ppbv)	Group C (ppbv)	Group D (ppbv)	Group E (ppbv)	Group F (ppbv)
85.065	$C_5H_8OH^+$	C_5H_8O-type feature	Carbonyl-related	0.12	0.07	0.35	0.41	0.51	0.31
87.044	$C_4H_6O_2H^+$	Butanedione-like	Carbonyl-related	1.94	0.61	1.01	1.37	1.47	1.11
87.080	$C_5H_{10}OH^+$	Pentenol-like	Oxygenated	1.93	0.60	0.99	1.35	1.45	1.09
89.023	$C_3H_4O_3H^+$	$C_3H_4O_3$-type feature	Oxygenated	0.18	0.03	0.03	0.03	0.07	0.10
89.061	C_5H_8O	Unassigned feature	Unassigned	0.16	0.03	0.03	0.03	0.07	0.10
91.043	$C_3H_6O_3H^+$	Hydroxypropanoic acid-like	Acid-related	0.20	0.05	0.08	0.09	0.08	0.10
93.071	$C_7H_8H^+$	Toluene-like	Aromatic hydrocarbon-related	0.33	0.03	0.25	0.27	0.42	0.17
94.867	$C_2H_6S_2$	Sulfur-associated feature	Sulfur-associated	0.05	0.04	0.19	0.23	0.31	0.17
96.056	—	Unassigned feature	Unassigned	0.01	0.01	0.16	0.19	0.20	0.11
97.066	$C_6H_8OH^+$	C_6H_8O-type feature	Carbonyl-related	0.06	0.06	4.35	4.93	4.04	2.85
99.044	$C_5H_6O_2H^+$	Furanmethanol-like	Furan-related	0.00	0.01	0.06	0.05	0.07	0.03
99.080	$C_6H_{10}OH^+$	Hexenal-like	Carbonyl-related	0.41	0.02	0.11	0.12	0.17	0.09
101.024	$C_4H_4O_3H^+$	$C_4H_4O_3$-type feature	Oxygenated	0.17	0.08	0.33	0.30	0.46	0.25
101.096	$C_6H_{12}OH^+$	Hexanal-like	Carbonyl-related	0.10	0.08	0.32	0.30	0.45	0.26
103.039	$C_4H_6O_3H^+$	Acetoacetic acid-like	Acid-related	0.05	0.03	0.01	0.04	0.07	0.03
105.037	$C_4H_8OSH^+$	Methional-like	Sulfur-associated	0.32	0.15	0.09	0.13	0.15	0.12
107.051	$C_7H_6OH^+$	Benzaldehyde-like	Carbonyl-related	0.55	0.31	0.06	0.09	0.31	0.03
108.085	$C_7H_9NH^+$	Benzylamine-like	Nitrogen-associated	1.02	2.24	0.64	1.45	1.54	1.58
109.102	$C_8H_{12}H^+$	$C_8H_{13}^+$ feature	Hydrocarbon-related	0.17	0.33	0.29	0.37	0.54	0.38
111.047	$C_6H_6O_2H^+$	Acetylfuran-like	Furan-related	0.32	0.05	0.82	0.97	1.02	0.60
112.076	$C_6H_9NOH^+$	C_6H_9NO-type feature	N/O-containing	0.01	0.01	0.09	0.11	0.13	0.06
115.075	$C_6H_{10}O_2H^+$	Caprolactone-like	Oxygenated	0.02	0.01	0.05	0.05	0.06	0.03
115.112	$C_7H_{14}OH^+$	$C_7H_{14}O$-type feature	Carbonyl-related	0.02	0.01	0.06	0.06	0.06	0.04
121.065	$C_8H_8OH^+$	C_8H_8O-type feature	Oxygenated aromatic	0.86	0.64	0.08	0.15	0.19	0.12
127.112	$C_8H_{14}OH^+$	$C_8H_{14}O$-type feature	Carbonyl-related	0.09	0.18	0.15	0.19	0.28	0.20
129.127	$C_8H_{16}OH^+$	$C_8H_{16}O$-type feature	Carbonyl-related	0.01	0.02	0.03	0.05	0.05	0.03
137.132	$C_{10}H_{16}H^+$	Monoterpene-like	Terpene-related	0.00	0.01	0.08	0.14	0.15	0.08
143.143	$C_9H_{18}OH^+$	Nonanal-like	Carbonyl-related	0.05	0.04	0.06	0.12	0.12	0.08
153.127	$C_{10}H_{16}OH^+$	Decadienal-like	Carbonyl-related	0.00	0.00	0.01	0.02	0.02	0.01
157.157	$C_{10}H_{20}OH^+$	Decanal-like	Carbonyl-related	0.05	0.05	0.07	0.09	0.16	0.10
160.994	—	Unassigned feature	Unassigned	0.00	0.00	0.01	0.01	0.01	0.00
177.161	$C_8H_{20}N_2O_2H^+$	Unassigned feature	Unassigned	0.03	0.01	0.05	0.05	0.08	0.08
195.174	$C_{13}H_{22}OH^+$	Geranylacetone-like	Carbonyl-related	0.02	0.00	0.04	0.04	0.06	0.05

Note: Values are arithmetic means of three independent cans (n = 3) and are semi-quantitative ppbv estimates. Group A, raw krill meat; Group B, pretreated, filled, exhausted, and vacuum-sealed but non-retorted samples; Groups C–F, F_0-equivalent samples retorted at 110, 115, 118, and 121 °C, respectively. Putative formulas and annotations are based on the PTR-ToF-MS data-processing workflow, PTR ion chemistry, and

literature information; the suffix “-like” does not denote definitive structural identification. Features with ambiguous structural assignments are reported at formula-, family-, or unassigned-feature level. Values shown as 0.00 are rounded semi-quantitative responses and should not be interpreted as validated non-detects or below-LOQ results.
